# Development of a compact cavity BPM for real-time monitoring of clinical proton beams at HUST-PTF

**DOI:** 10.3389/fonc.2025.1508361

**Published:** 2025-06-27

**Authors:** Jiqing Li, Yuexin Lu, Jiapeng Li, Jian Wang, Zhengzheng Liu, Oleg Meshkov, Jinfeng Yang, Kuanjun Fan

**Affiliations:** ^1^ State Key Laboratory of Advanced Electromagnetic Technology, Huazhong University of Science and Technology, Wuhan, China; ^2^ Budker Institute of Nuclear Physics SB RAS, Novosibirsk, Russia; ^3^ Institute of Scientific and Industrial Research, Osaka University, Osaka, Japan

**Keywords:** proton therapy, beam position monitor, cavity, shunt impedance, off-center, dielectric loading

## Abstract

**Introduction:**

To ensure the safety and efficacy of precise proton therapy, real-time and non-intrusive monitoring of the clinical beam position is essential. However, in cyclotron-based proton therapy facilities, clinical proton beams with low repetition frequency and exceptionally low intensity due to the Energy Selection Systems (ESS), pose considerable challenges for accurate online beam diagnostics. Conventional non-interceptive beam diagnostic devices lack the sensitivity required to detect such weak beams with sufficient precision.

**Methods:**

This paper presents an innovative solution to this challenge: an off-centerrectangular cavity Beam Position Monitor (BPM) with dielectric loading. This novel design achieves remarkable position sensitivity while maintaining compact transverse dimensions of 500×250×100 mm.

**Results:**

A prototype of this cavity has been fabricated and tested offline. Experimental results demonstrate that, within the clinical treatment energy range, the BPM achieves minimum beam position measurement sensitivities of 0.49 nV/mm at 70 MeV and 17.12 nV/mm at 230 MeV. In addition to enabling online beam position monitoring without disturbing the beam path, which ensures real-time beam orbit feedback correction with submillimeter stability (± 0.5 mm).

**Discussion:**

In addition to monitoring beam positions for precise control of the beam orbit, the BPMs could serve additional functions to enhance proton therapy—such as enabling beam energy verification through the phase of the BPM signal.

## 1 Introduction

**Figure 1:**
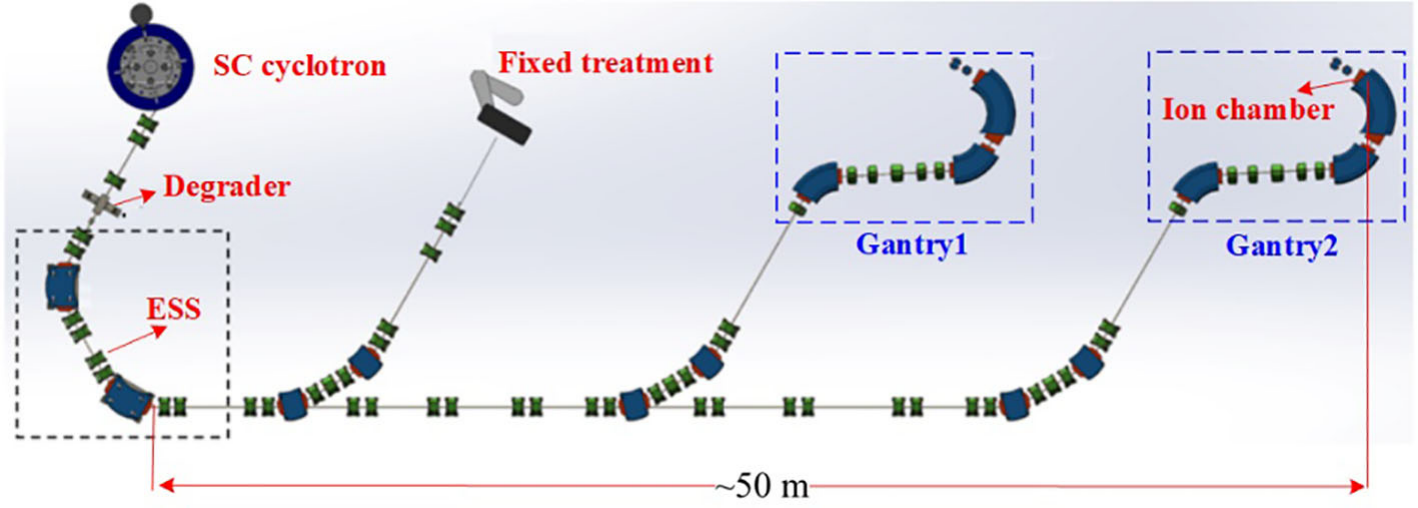
Schematic layout of HUST-PTF.

Proton therapy is becoming the preferred treatment globally because of its precision in targeting tumors (1–3). The Proton Therapy Facility (PTF) at Huazhong University of Science and Technology (HUST) is currently under commissioning (4–6). As depicted in **Figure 1**, this facility includes a 250 MeV superconducting cyclotron, a beam transport line, two rotating gantries, and a fixed treatment room. **Table 1** lists the key parameters. A Graphite-based energy degrader is utilized to modify the cyclotron’s fixed energy to a variable range from 70–230 MeV (6). The ESS integrates collimators with a double bend achromat (DBA) dispersive-free section, discriminatively transmitting protons within target energy windows while suppressing high-scatter-angle particles at the expense of reduced transmission. This dual-stage filtering mechanism ensures beam parameters comply with rigorous clinical specifications (energy spread<1%, emittance<5 π mm·mrad). For instance, at an energy of 230 MeV, only 8.34% of the scattered beam is transmitted through the ESS; this transmission efficiency decreases to 0.07% when the beam energy is reduced to 70 MeV (6). Once they have passed through the ESS, the protons travel approximately 50 meters along a beam transport line before reaching the nozzle, where ion chambers are for beam monitoring.

**Table 1:** Key parameters of HUST-PTF.

Parameters	Value
Extraction beam energy	250 MeV
Beam current from the cyclotron	>500 nA
Clinical beam energy	70–230 MeV
Beam current after the ESS	0.35–5 nA
Cyclotron RF frequency	73 MHz

However, in practical beam transport lines, various factors, such as errors in the dipole magnetic field and magnet positioning, can cause protons to deviate from their intended path, resulting in orbit distortions. These deviations can reduce the precision of proton beam dose delivery. Implementing real-time orbit correction can mitigate this and maintain the proton beam’s optimal trajectory. Precise control requires rapid, accurate, online measurements of the beam’s intensity and position throughout the transport line.

However, conventional beam diagnostic devices cannot detect proton beams of approximately 0.35 nA, necessitating the use of interceptive diagnostic devices (7), such as fluorescent screens and Faraday cups, for commissioning in a section-by-section manner. Although ionization chambers (8, 9) are highly sensitive for measuring beam current and position, their scattering interactions can degrade beam quality, especially when multiple ionization chambers are employed along the beam transfer line.

Clinical treatment could benefit from continuous monitoring of proton beams throughout their entire journey rather than merely measured at the beamline’s beginning or end. To achieve non-interceptive tracking, we can measure either the image current (10, 11) or the excited electromagnetic (EM) fields within an RF cavity (12, 13). Image current devices often lack the necessary sensitivity to monitor these beams.

In contrast, cavity-type beam diagnostic devices (14–16) (including Beam Current Monitors (BCMs) and cavity Beam Position Monitors (BPMs)) offer an advantage as cavity resonance improves the signal-to-noise ratio. Traditional cavity BPMs, often using a pillbox structure, are widely used in electron accelerators. Operating in the C-X band (3–9 GHz), their resonant cavities can be designed with compact dimensions. However, using conventional cavity BPMs for the HUST-PTF transport line poses significant challenges. First, the proton beam’s microstructure, tied to the cyclotron’s operating frequency (73 MHz), is too low for traditional cavity BPMs. Resonating at this frequency would require impractically large transverse dimensions. Second, conventional cavity BPMs operate in a dipole mode with low beam excitation efficiency. For the clinical proton beam currents around 0.35nA, the signal cannot be detected from the noise.

In order to overcome the above challenges faced in traditional models, recently, innovative designs in cavity BCMs and BPMs have been developed for the HUST-PTF. A novel prototype of a compact cavity BCM that demonstrated the feasibility of reducing cavity size through dielectric loading has been reported (17). This paper proposes the use of an off-center rectangular cavity BPM, also employing dielectric loading, which operates in the fundamental mode.

## 2 Cavity BPM pick-up principle

### 2.1 Working modes

An ideal cavity is an empty space enclosed by conducting walls that allow electromagnetic (EM) fields to be generated and confined when charged bunches pass longitudinally through it. A cavity designed to excite only transverse magnetic (TM) modes will have a vanishing longitudinal component of the magnetic field and an electric field aligned with the beam path. This alignment provides a strong coupling between the cavity and the beam. The electromagnetic field produced contains information on the beam, which can be measured.

**Figure 2:**
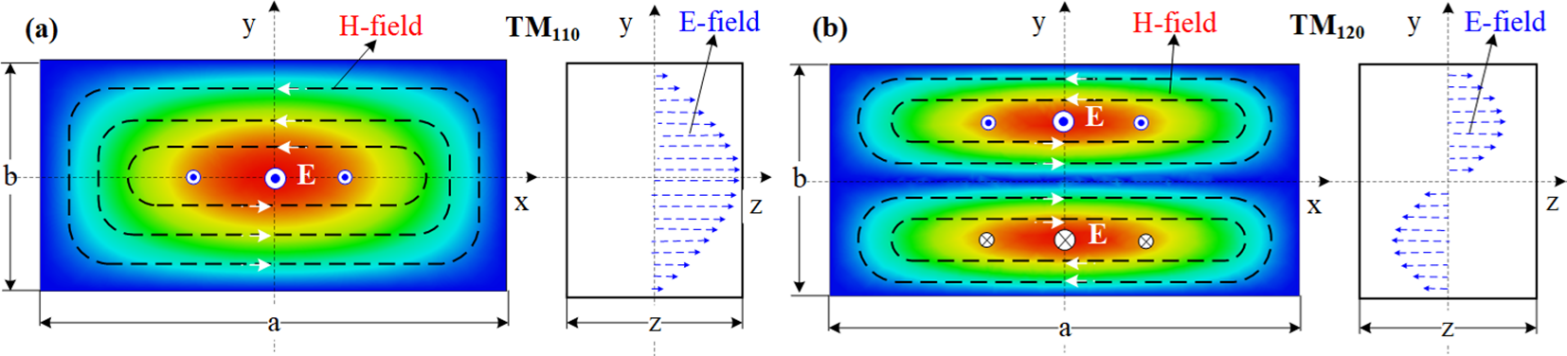
EM field patterns of **(a)** monopole and **(b)** dipole modes in a rectangular cavity.

This paper presents the use of a rectangular cavity for beam position measurement. A rectangular cavity is easier to fabricate compared to a cylinder. It provides sufficient beam position measurement accuracy and can measure horizontal or vertical positions separately. This cavity is designed with dimensions of 500×250×100 mm, and beam holes are incorporated into the walls to allow for coupling with the beams. In the case of a rectangular cavity, the monopole and dipole modes are used. **Figure 2** shows the electromagnetic field modes. The monopole mode TM110 (**Figure 2a**) can be easily excited and has the strongest electric field in the center of the cavity. The excited electric field is directly proportional to the bunch charge and is concentrated almost exclusively around the center region, making it ideal for applications such as beam acceleration or intensity detection. In contrast, the dipole mode TM120 (**Figure 2b**) has a frequency that is close to that of the monopole mode. Its electromagnetic fields are mirror symmetric and increase linearly in the y direction near the center of the cavity. Since TM120 mode exhibits linear field growth along the y-axis with x-independence, this configuration inherently enables spatial decoupling of x-directional contributions from y-position monitoring. However, to simultaneously achieve x-directional beam position measurement, an orthogonally positioned identical structure is required to establish independent field monitoring channels.

### 2.2 Working frequency

The eigenfrequency of TM modes only takes discrete values determined exclusively by the dimensions of the cavity. The resonant frequency of the TMmnp mode in a rectangular cavity is given by the following Equation:


(1) $$\begin{array}{c} f_{rec,mnp}=\frac{c}{2\pi }\sqrt{{\left(\frac{m\pi }{a}\right)}^{2}+{\left(\frac{n\pi }{b}\right)}^{2}+{\left(\frac{p\pi }{L}\right)}^{2}} \end{array}$$

In this Equation, *m*, *n*, and *p* represent the mode indices, while *a*, *b*, and *L* are the cavity dimensions along the x, y, and z axes, respectively. Here, *c* denotes the speed of light in a vacuum.

The resonant frequency of the TMmnp decreases as the transverse dimensions increase. In periodic multi-bunch diagnostics, cavities are typically tuned to an integer multiple of the bunch microstructure frequency. In the HUST - PTF, the microstructure frequency is 73 MHz, which is determined by the operating frequency of the superconducting cyclotron. To downsize the cavity-type BPM, its operating frequency is set at 146 MHz, precisely twice the repetition frequency of the proton microstructure.

### 2.3 Shunt impedance

To estimate the resolution expected from the cavity BPMs, further analysis of the energy exchange between the beam and the cavity is essential. This interaction is influenced by both the cavity’s geometry and the proton microstructure properties, which are characterized by shunt impedance (a critical parameter). Higher shunt impedance results in a higher sensitivity to the beam position. The normalized shunt impedance for each operational mode serves as a critical parameter for this assessment, which is defined as the shunt impedance divided by the unloaded quality factor (*R_sh_*/*Q*_0_) (12, 15, 18), expressed mathematically as Equation 2:


(2) $$\begin{array}{c} \frac{R_{sh}}{Q_{0}}=\frac{{\left|V_{ext}\right|}^{2}}{\omega U} \end{array}$$

Where *V*_ext_ represents the excited voltage across the cavity, *ω* denotes the resonance frequency, and *U* is the storage energy within the cavity.

For example, if a rectangle cavity works in the TM110 mode, the normalized shunt impedance in the center region (y=0) is defined as Equation 3:


(3) $$\begin{array}{c} {\left(\frac{R_{sh}}{Q_{0}}\right)}_{rec,110}=\frac{8LT^{2}}{\omega \epsilon_{0}ab}\cos^{2}\left(\frac{\pi }{a}x\right) \end{array}$$

Here, a, b, and L denote the cavity dimensions along the x, y, and z axes, respectively; T represents the transient factor; and ϵ_0_ is the permittivity of free space. Increasing the normalized shunt impendence increases the beam position monitor signal.

## 3 Design and optimization of the BPM cavity

The signal-to-noise ratio (SNR) increases with the beam’s energy loss to the operational mode and external coupling efficiency. Conventional cavity BPMs with no dielectric are not suitable for monitoring the clinical proton of HUST-PTF due to two major challenges. Firstly, as clearly shown by Equation 1, the low operating frequency (146 MHz) results in a large transverse size. Secondly, conventional cavity BPMs utilizing dipole modes exhibit significantly weaker electric field intensity compared to the fundamental mode. This inherent limitation results in weak signal generation during low-intensity proton beam detection, consequently compromising SNR.

### 3.1 Off-center rectangular cavity works in monopole mode

**Figure 3:**
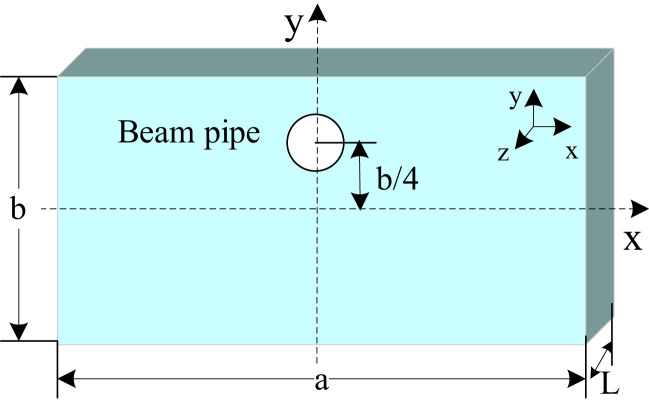
Rectangular cavity with off-center beam pipe.

To address these challenges mentioned above, the proposed solution is a novel rectangular cavity BPM with an off-center beam pipe working in monopole mode with dielectric loading, as shown in **Figure 3**. Cavity BPMs traditionally use secondary operation modes (dipole modes). At the same operating frequency, their transverse dimensions are larger than those of cavities operating with fundamental modes. Equation 1 shows that when the operating frequency is 146 MHz, the transverse dimensions of a rectangular resonant cavity (with an aspect ratio of 2:1) working in the TM120 mode are 4236 mm × 2118 mm, which is too large for practical applications. Furthermore, the longitudinal electric field excited in the dipole modes is weaker than that of fundamental modes. This results in a lower signal amplitude being extracted from the TM120 mode, which, in turn, limits sensitivity. In contrast, operating in the TM110 mode reduces the transverse dimensions of the cavity to 2298 mm × 1149 mm, a 71% reduction in the transverse area.

GSI proposed using the fundamental mode TM110 in a rectangular cavity to measure the beam’s transverse position to improve the cavity BPM’s sensitivity and simultaneously reduce its transverse dimensions (19, 20). For a rectangular resonant cavity with dimensions a, b, and L along the transverse length, width, and longitudinal height, respectively, the longitudinal normalized shunt impedance expression for the TM110 mode is given by Equation 4:


(4) $$\begin{array}{c} \frac{R_{sh}}{{\left(Q_{0}\right)}_{rec,110}}=\frac{8LT^{2}}{\omega \epsilon_{0}ab}\cos^{2}\left(\frac{\pi }{a}x\right)\cos^{2}\left(\frac{\pi }{b}y\right) \end{array}$$

In the region of the central axis of the rectangular cavity, the normalized shunt impedance for the TM110 mode remains essentially unchanged. However, if the center position of the beam pipe is shifted to the middle of the short semi-axis of the rectangular cavity (see **Figure 3**), then near the center of the beam pipe where *x*=*x*’ and *y*=1/4*b*+*y*’ (where *x*’ and *y*’ represent the displacement of the beam from the center of the beam pipe), substituting the expressions for *x* and *y* into the normalized shunt impedance equation yields Equation 5:


(5) $$\begin{array}{c} \frac{R_{sh}}{{\left(Q_{0}\right)}_{rec,110}}=\frac{4LT^{2}}{\omega \epsilon_{0}ab}\cos^{2}\left(\frac{\pi }{a}x^{\prime }\right){\left[cos\left(\frac{\pi }{b}y^{\prime }\right)-sin\left(\frac{\pi }{b}y^{\prime }\right)\right]}^{2} \end{array}$$

for y’<<*b*,


(6) $$\begin{array}{c} \frac{R_{sh}}{{\left(Q_{0}\right)}_{rec,110}}=\frac{4LT^{2}}{\omega \epsilon_{0}ab}\cos^{2}\left(\frac{\pi }{a}x^{\prime }\right)\left(1-sin\left(\frac{2\pi }{b}y^{\prime }\right)\right) \end{array}$$

Equation 6 clearly shows that modifying the beam pipe’s position makes *R_sh_* dependent on *y*’. This ensures accurate beam position measurements while maintaining the enhanced output signal characteristics of the TM110 mode.

According to Equation 6, when the center of the beam pipe is aligned with the midpoint of the short semi-axis of the rectangular cavity, the normalized shunt impedance exhibits minimal sensitivity to small displacements of the beam in the x-direction. In contrast, displacements in y primarily affect the normalized shunt impedance.

**Figure 4:**
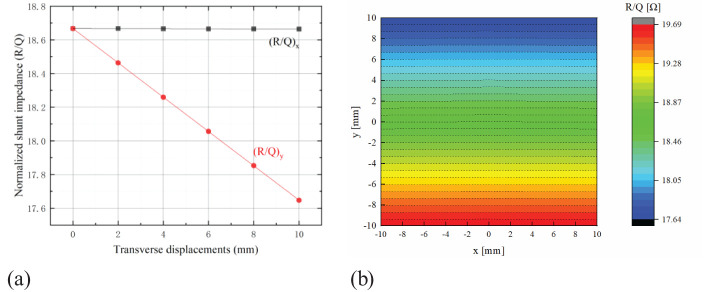
Normalized shunt impedance within the beam pipe. **(a)** CST Simulated data of **Tables 1**, **2** **(b)** 2D map of normalized shunt impedance from the CST simulation.

For a cavity length of 100 mm, we have theoretically calculated and simulated the normalized shunt impedance values for various transverse positions near the center of the beam pipe using CST Microwave Studio. The results are presented in **Tables 2**, **3**, together with the results from Equation (6). Notably, the Normalized Shunt Impedance (NSI) values derived from Equation (6) agree within 0.04% with CST simulations, with discrepancies remaining within 0.1%. **Figure 4a** shows the calculation results.

**Table 2:** Calculated [Equation (6)] and CST simulation results of normalized shunt impedance for different beam offset in *x*-direction (beam offset in *y*-direction is fixed to 0).

R/Q	Offset in *x*-direction					
	0 mm	2 mm	4 mm	6 mm	8 mm	10 mm
Theoretical (Ω)	18.660	18.660	18.660	18.659	18.658	18.657
CST Simulation (Ω)	18.667	18.667	18.666	18.665	18.665	18.664
Discrepancies	0.04%	0.04%	0.03%	0.03%	0.04%	0.04%

**Table 3:** Calculated (Equation (6)) and CST simulation results of normalized shunt impedance for different beam offset in *y*-direction (beam offset in *x*-direction is fixed to 0).

R/Q	Offset in *y*-direction					
	0 mm	2 mm	4 mm	6 mm	8 mm	10 mm
Theoretical (Ω)	18.660	18.456	18.252	18.048	17.844	17.640
CST Simulation (Ω)	18.667	18.463	18.259	18.056	17.853	17.647
Discrepancies	0.04%	0.04%	0.04%	0.04%	0.05%	0.04%

The CST simulation shows that *R_sh_* is independent of x for up to ±10 mm changes in x direction (**Figure 4b**). Consequently, employing two orthogonally placed rectangular cavities can be used to measure the beam’s transverse position.

### 3.2 Dielectric loaded off-center cavity geometry optimization

While the off-center rectangular cavity operating in the TM110 mode provides smaller transverse dimensions compared to traditional designs, its dimensions still remain large. Leemann (21) proposed that the resonant frequency can be lowered by placing two metal rods along the axis, with gaps between the rods and the cavity wall on one side. The fundamental principle of this methodology lies in its concentration of the resonant electric field within the interstitial regions between the rod elements, thereby inducing a dual effect of enhancing cavity capacitance while reducing resonant frequency.

**Figure 5:**
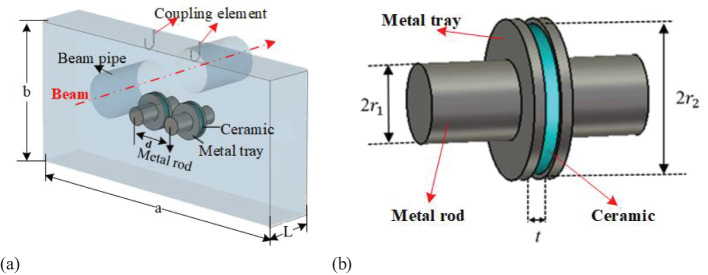
Off-center rectangular cavity model filled with dielectric. **(a)** Structure of the cavity **(b)** Metal rod, tray, and dielectric.

However, the aforementioned approach fails to achieve adequate field concentration within the gaps, thereby restricting the possibility of further dimensional reduction in the cavity structure. Given that the electric field of the TM110 mode is most intense along the central axis of the cavity, introducing dielectric material can further lower the resonant frequency (22, 23). As illustrated in **Figure 5**, two pairs of metal rods are connected to metal trays designed to hold dielectric disks. Additionally, the resonant frequency can be finely tuned by adjusting the dimensions of the metal rods or trays.

Two crucial parameters significantly influence performance: 1) the gradient of the normalized shunt impedance along the beam pipe, which directly affects the BPM’s positional resolution, and 2) the frequency sensitivity, i.e. the change in frequency per change in the thickness of the dielectric disk, denoted as *t*. Following optimization, the cavity’s transverse dimensions are established at 500 mm×250 mm, with a longitudinal length of 85 mm. The chosen dielectric material is 99.5% pure alumina ceramic, which has a dielectric constant of ϵ= 9.8 and exhibits a low dielectric loss of less than 0.0001. Since the cavity does not include a tuner, it is critical to maintain the frequency sensitivity within 0.5 MHz per 0.1 mm to minimize frequency shifts. After optimization, the final parameters have been determined as follows: *r*_1_ = 15 mm, *r*_2_ = 28 mm, and *t* = 11.1 mm.

### 3.3 Beam induced electromagnetic field within cavity

**Figure 6:**
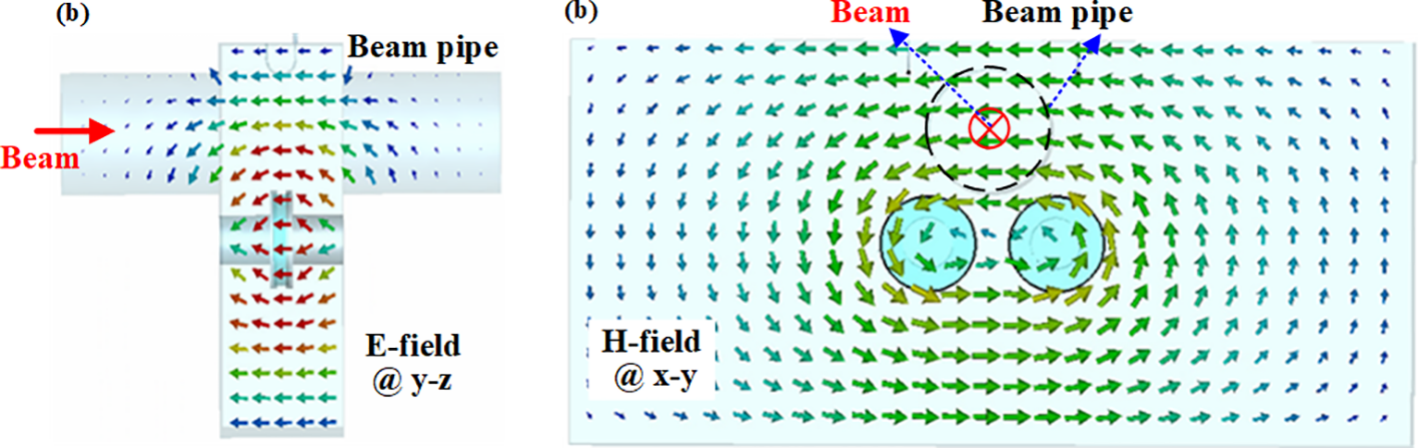
Electromagnetic field distribution of operation TM110 mode **(a)** E-field @ side view **(b)** H-field @ front view.

The electromagnetic field behavior excited by the passing beams within the cavity was analyzed using CST software, as depicted in **Figure 6**. **Figure 6a** illustrates the electric field distribution in the y-z cross-section, while **Figure 6b** presents the magnetic field distribution in the x-y cross-section. Due to the presence of the dielectric disk, the electric field of the operating mode exhibits incomplete longitudinal concentration, with negligible components existing in the transverse direction, while the magnetic field is predominantly distributed transversely. Therefore, the operational mode of this dielectric-loaded off-axis rectangular cavity BPM can be categorized as a quasi-TM mode.

**Figure 7:**
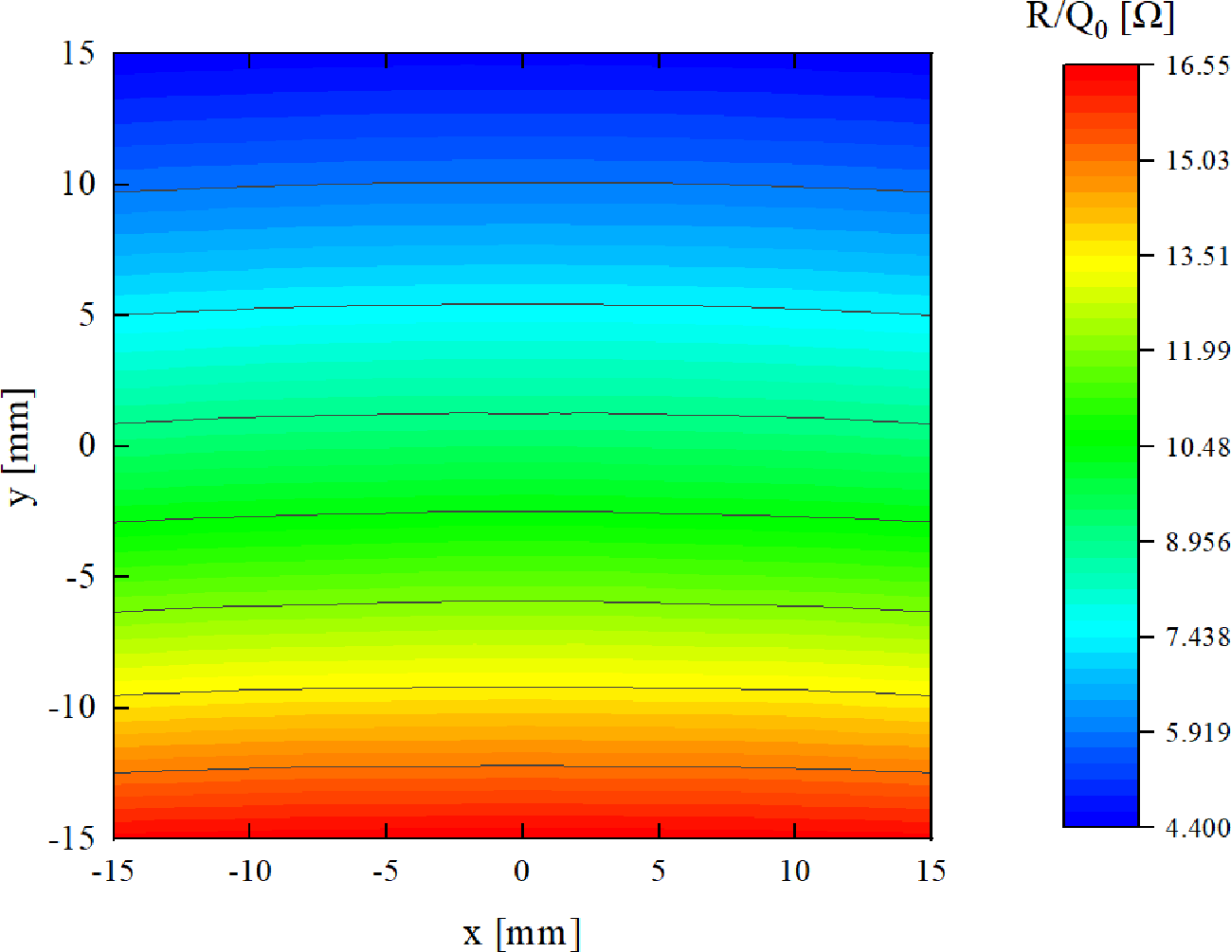
Normalized shunt impedance map within beam pipe center of ±15 mm with the cavity that has the dielectric.

**Figure 7** presents the map of the normalized shunt impedance (*R/Q*) near the center of the beam pipe. Within a ±15 mm range around the pipe’s center, the contour lines are approximately parallel to the x-axis. This observation indicates that when the beam moves laterally within this region, the amplitude of the BPM output signal is predominantly influenced by the beam’s displacement in the y direction, with minimal crosstalk between the x and y axes. The actual operation includes variations in pulse duration. However, the output signals of the BPMs primarily depend on the normalized shunt impedance. Consequently, these minor alterations in proton pulse structure only affect the average beam amplitude without compromising the positional information accuracy. Although the contours curve slightly at the edges of this region, potentially introducing some crosstalk, it is not anticipated to be a significant concern, as most protons are expected to traverse the central area where the contours are primarily straight. By normalizing for beam intensity, a pair of orthogonally arranged cavities can accurately determine the beam’s transverse position.

This paired BPM configuration allows for precise beam energy verification through phase analysis of the BPM signals. In the HUST-PTF facility, two critical proton energy parameters require continuous monitoring: the cyclotron’s nominal output energy (E_0_) and the clinical treatment energy (Ec).

Theoretically, the cyclotron’s design output energy remains invariant, serving as the baseline for subsequent degrader adjustments to achieve the prescribed proton energy for therapeutic applications. However, variations in accelerator operating conditions, particularly phase fluctuations in the RF accelerating field, may induce deviations in the cyclotron’s output energy. Such energy deviations can be quantitatively characterized by examining the phase correlation between the BPM signal and the cyclotron’s RF accelerating field.

During clinical treatment, the degrader controls the therapeutic proton beam energy through precise mechanical adjustment. Nevertheless, intrinsic limitations in the degrader’s calibration accuracy may lead to discrepancies between the achieved proton energy and the prescribed clinical value. The paired BPM system offers comprehensive real-time monitoring capability for the delivered clinical beam energy. Considering that therapeutic proton energies operate in the non-relativistic domain (70–230 MeV), their velocity exhibits a well-defined energy dependence. Continuous real-time energy monitoring is accomplished by precisely measuring the time-of-flight (TOF) interval between successive BPM signals as the proton beam traverses the paired detector system. For instance, with a 1 m center-to-center separation between BPMs, the measured TOF is 9.1049 ns at 70 MeV, decreasing to 5.5946 ns at 230 MeV, thereby providing reliable energy verification during therapeutic beam delivery.

## 4 Fabrication and offline testing of the cavity

### 4.1 Fabrication cavity

A prototype cavity was designed and fabricated for proof of principle, and stainless steel was chosen for the majority of its components to reduce cost. Using copper in constructing a real cavity BPM is expected to improve performance.

**Figure 8:**
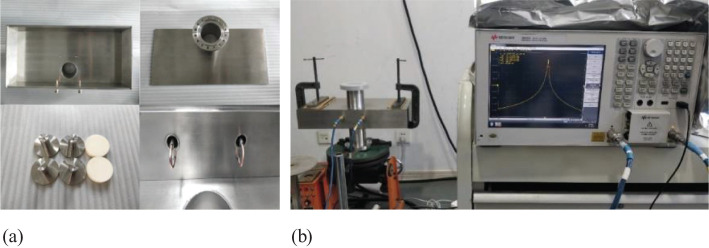
Pictures of the cavity before final assembly and measurement setup. **(a)** components of the cavity **(b)** measurement setup.

To enable precise tuning of the resonance frequency, the cavity was initially designed with a resonant frequency of 147.45 MHz, and its key components were intentionally manufactured slightly oversized to accommodate fine adjustments. Following the machining of the essential components of the rectangular cavity, a temporary assembly was constructed using fixtures for measurement purposes, as depicted in **Figure 8**. A network analyzer was employed to measure critical parameters, including the resonant frequency. This arrangement facilitated the accurate assessment and optimization of the cavity’s performance before the final assembly was completed.

**Figure 9:**
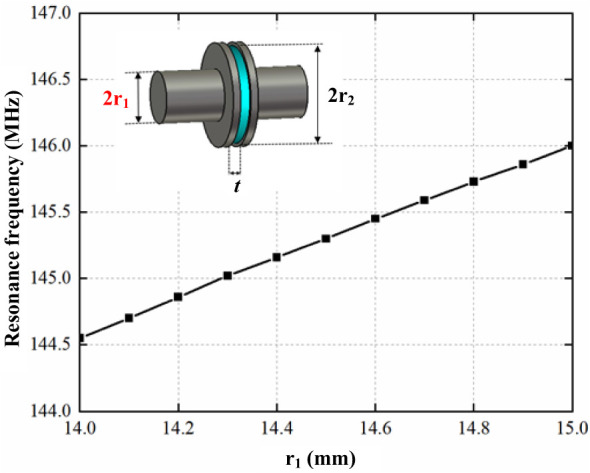
Effect of metal rod radius *r*_1_ on the resonant frequency.

To tune the resonant frequency to 146 MHz, we adjusted the small metal rods within the rectangular cavity. According to the CST simulation results presented in **Figure 9**, reducing the radius of these metal rods lowers the resonant frequency of the operating mode. Therefore, we decreased the rod radius by increments of 0.05 mm. Following each adjustment, we temporarily reassembled the cavity with fixtures to measure its resonant frequency. Once the frequency consistently stabilized at 146 MHz, we welded the cavity to ensure stability and performance.

**Figure 10:**
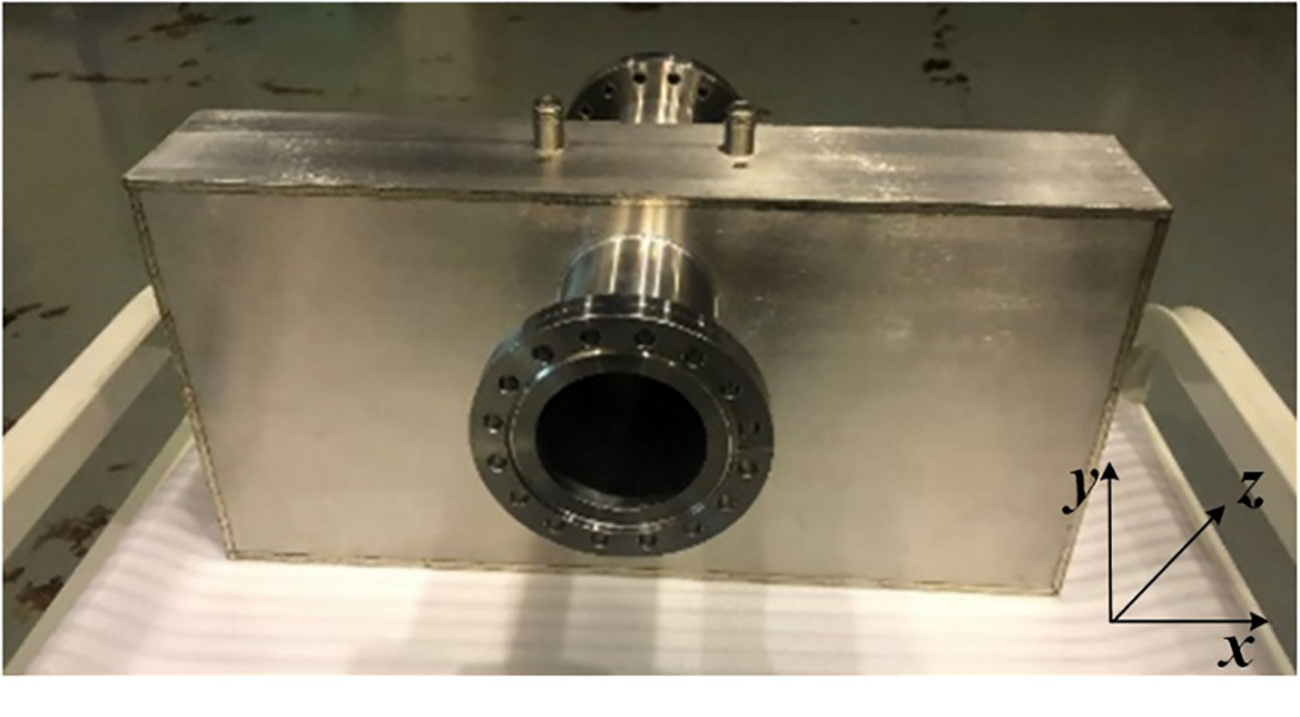
Picture of the assembled rectangular cavity (dimensions: *x*×*y*×*z*=500×250×85 mm).

**Figure 11:**
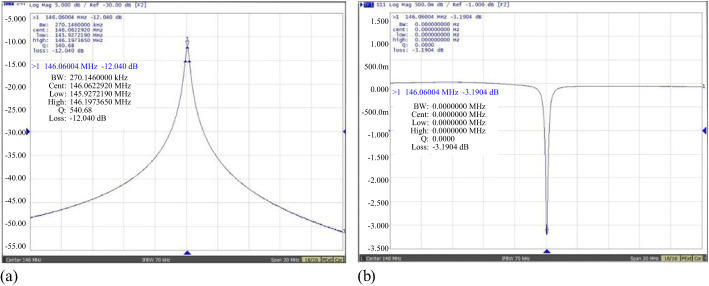
S-parameter measurement of the cavity. **(a)** S_21_ measurements **(b)** S_11_ measurement.

**Figure 10** depicts the welded rectangular cavity BPM, now operating at the frequency of 146.06 MHz. **Figure 11** displays the results of the S-parameter measurements. The unloaded quality factor Q_0_ of the cavity was measured at 780.2, exhibiting a 14.6% deviation from the designed specification of 913.4. Meanwhile, the external quality factor Q_ext_ demonstrated superior performance with a measured value of 1761, surpassing the design target by 17.4%. These measurement discrepancies primarily originate from machining tolerances exceeding acceptable limits, manifesting as microscopic surface irregularities on the internal wall surfaces. Notably, since this prototype cavity is primarily intended for proof-of-concept validation, the machining precision and material selection were not rigorously controlled to reduce development costs. Consequently, certain measurement parameters exceeded the initially specified tolerance limits but remained within permissible operational margins, without compromising the cavity’s functional integrity.

### 4.2 Measurement of normalized shunt impedance

**Figure 12:**
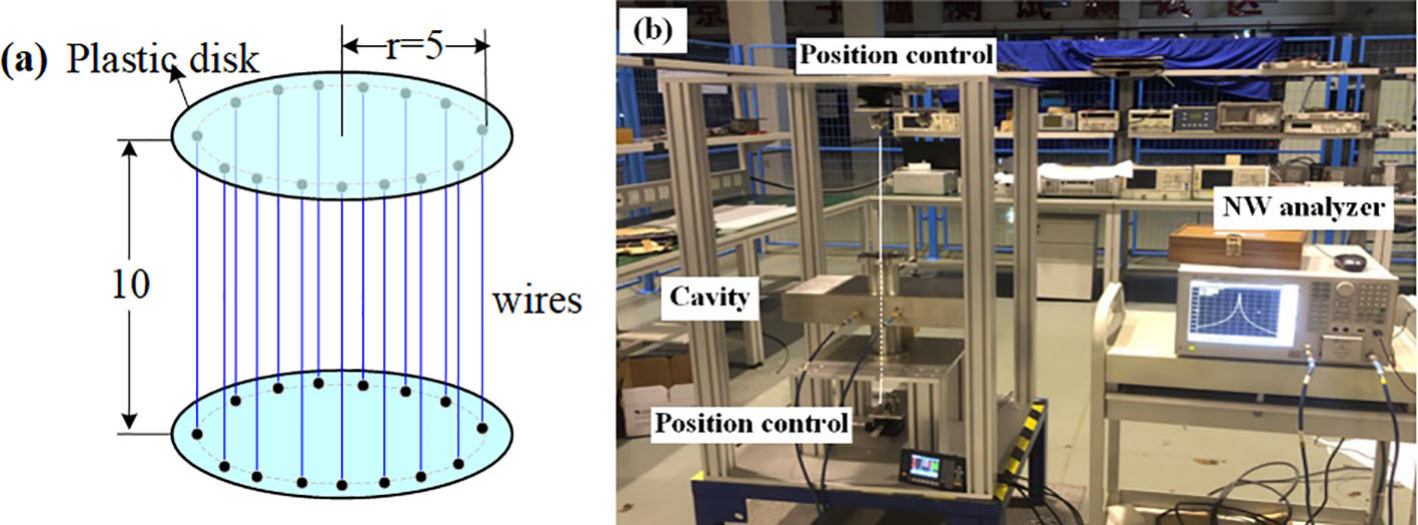
Normalized shunt impedance measurement using birdcage perturbance. **(a)** birdcage schematic **(b)** measurement setup (birdcage inside the cavity not depicted in the figure; its position can be precisely adjusted by up and down position controllers).

The output signal amplitude of the BPM cavity is primarily determined by the normalized shunt impedance along the beam direction. Therefore, accurate measurement of this impedance is critical for evaluating the cavity’s performance characteristics. However, the introduction of dielectric material at the cavity’s center introduces electromagnetic field components in both longitudinal and transverse directions, which significantly compromises the measurement accuracy. Traditional cylindrical and spherical perturbators exhibit inherent limitations in achieving directional electric field modulation along the axial axis. To address this issue, a birdcage perturbation device (24) has been engineered to induce controlled perturbations in the axial electric field. This device employs metal wires to specifically perturb the electric field along their alignment, enhancing the measurements’ resolution and sensitivity. Extensive details regarding the operational mechanism of the device are elaborated in the cited works (25). **Figure 12a** illustrates the birdcage perturbation device, consisting of a plastic disk with a 5.5 mm radius to hold wires (26, 27). Metal wires with a 0.5 mm diameter and 10 mm length are symmetrically positioned around a circumference that measures 5 mm in radius surrounding the disk. After calibrating, the number of wires was set to 12. This configuration induces controlled perturbations in the cavity’s electric field, enabling high-precision shunt impedance measurements.

**Figure 13:**
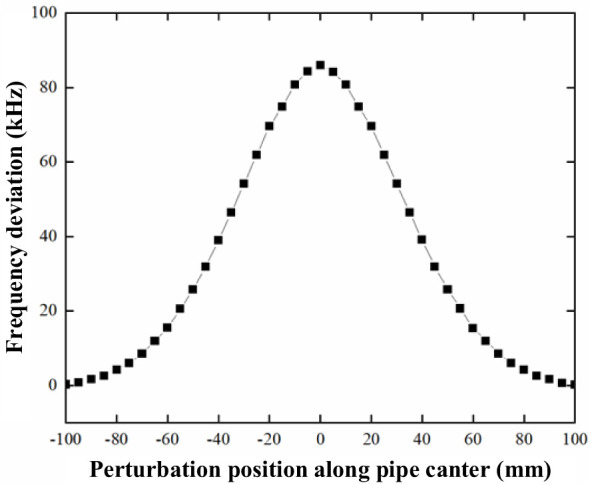
Measured frequency deviation with respect to the distance of the birdcage along the beam pipe.

An automated test bench has been built to measure the electric field within the cavity. This bench incorporates the birdcage perturbation device, which is driven by a stepper motor to traverse along the central axis of the beam pipe, as depicted in **Figure 12b**. The change in frequency directly depends on the distance of the birdcage along the beam pipe, and the resulting deviation frequency, Δf, is recorded at various positions. For enhanced accuracy, ten measurements are taken at each position, and the average deviation frequency at the reference position is calculated to minimize measurement uncertainty, as shown in **Figure 13**. The calculated normalized shunt impedance at the beam pipe position was found to be 9.45 Ω.

### 4.3 Beam simulation measurement

**Figure 14:**
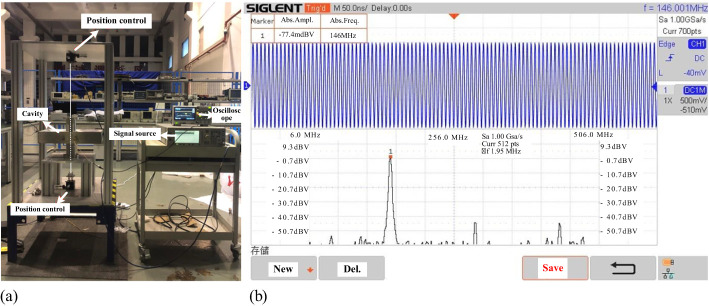
Beam measurements with signal introduced by a wire. **(a)** Measurement setup **(b)** Measured signal.

**Figure 15:**
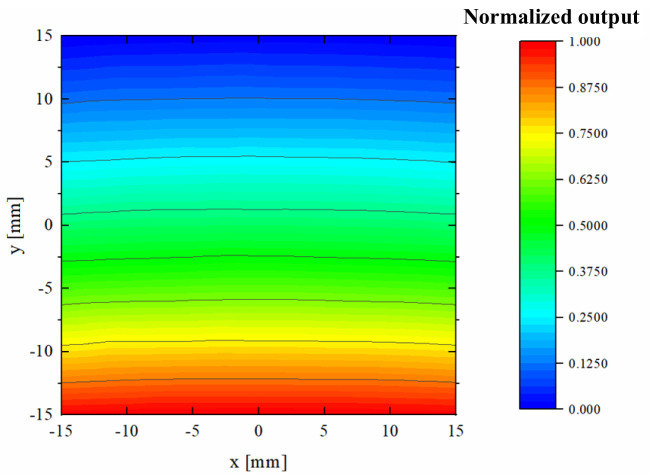
Map of measured normalized output signal relative to the transverse positions of wire.

Using the wire displacement method, beam simulation results for the cavity were conducted on the automated test bench, as illustrated in **Figure 14**. Initially, a single thin metal wire was stretched through the center of the beam pipe. A 146 MHz sine-wave signal was then introduced into the cavity from an RF signal generator to simulate the transient response of the proton beam transition. The resulting induced voltage signal from the pickup within the cavity was subsequently extracted to an oscilloscope, allowing observation of the EM field distribution in both the time domain and frequency domain. The transverse position of the metal wire is scanned at constant RF signal generator output. The amplitude of the RF signal generator’s output is maintained constant while the transverse position of the metal wire, relative to the center of the beam pipe, is varied to simulate different transverse beam offsets. The wire is maneuvered over a range of ±15 mm with a displacement accuracy of less than 0.005 mm. For each position of the metal wire, the amplitude of the output signal is recorded. The measured results are depicted in **Figure 15**, exhibiting excellent spatial correlation with the two-dimensional distribution of the cavity’s normalized shunt impedance obtained via computational simulations.

### 4.4 Output signal dependence on energy

In practice, the dielectric-loaded off-center rectangular cavity BPM is strategically positioned immediately downstream of a reference cavity BCM, so the output of the BPM can be normalized by the BCM signal, assuming the same beam current traverses both devices. Our offline test data encompassed beam positions in the range of x= ± 10 mm and y= ± 10 mm, which is more than the maximum expected variation in the beam position.

The energy of the clinical proton beams typically spans from 70 MeV to 230 MeV, which impacts the output signal of the BPM used to track the beam’s position. Simulations show that a 70 MeV proton beam exhibits a transit time factor of 0.962, and the beam current is 0.35 nA in our machine. As it passes through the cavity BPM at different lateral positions, the output signal amplitude varies between 18.68 nV and 28.52 nV. The position sensitivity of the BPM at this energy is 0.49 nV/mm. At 230 MeV, the simulation shows a transit time factor of 0.985, and the beam current is 5 nA in our machine. This increased energy and current result in a larger output signal amplitude ranging from 650.79 nV to 993.13 nV at different lateral positions. The position sensitivity at this energy level is higher, at 17.12 nV/mm. These variations in output signal amplitude and position sensitivity at different proton beam energies need to be characterized for the proper operation of the BPM.

**Figure 16:**
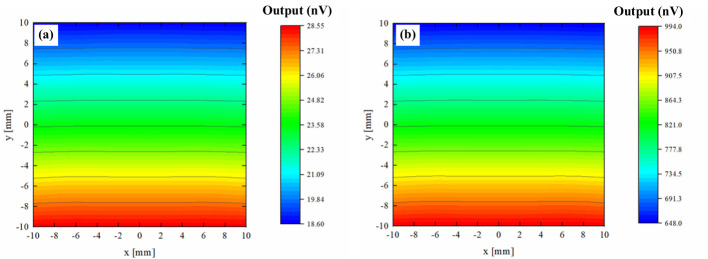
Comparison of measured output signal from the cavity BPM. **(a)** E=70 MeV, I=0.35 nA, **(b)** E=230 MeV, I=5 nA.

**Figure 16** illustrates the simulated amplitude of the BPM output signal at various transverse positions for proton beams at both 70 MeV and 230 MeV. This data highlights the critical need for energy-specific calibration of the BPM.

### 4.5 Crosstalk between x and y

The x-y directional coupling characteristics of the BPM output signal were experimentally studied. The experiments employed a stationary measurement approach: First, the beam position was fixed along the y-axis, and the output signal at x=0 was established as the reference baseline. We acquired normalized voltage response curves for x-directional displacements (Δx=−10~+10 mm) relative to the reference point. By calculating the normalized relative deviation δ(Δx)=|V(Δx)−V_0_|/V_0_×100% for each displacement, we quantified the coupling effect. The use of orthogonally aligned BPMs enables concurrent measurement of beam signals in the x- and y-directions, resulting in a consistent x-y coupling variation along the x-axis.

**Figure 17:**
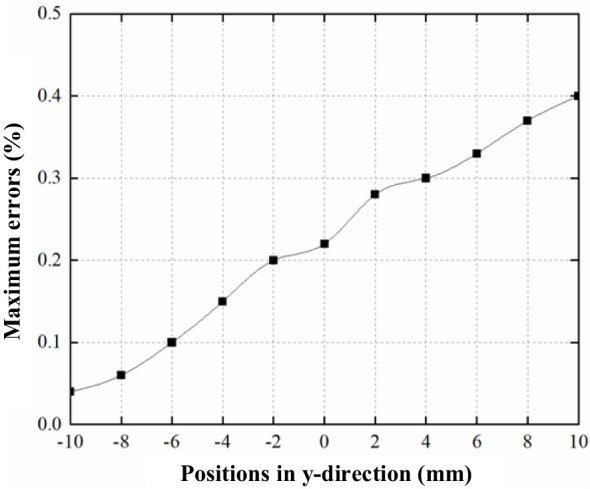
The maximum change in measured signal (%) over +/- 10 mm comparing to the central axis relative to y-position due to crosstalk.

**Figure 17** shows that as the beam moves along the y-axis, there is an increase in the coupling between the x and y directions. However, within ±10 mm of the center of the pipe—a typical range of operation—the output signal error due to this x-y coupling remains within 0.5%. This level of error is acceptable, especially when considering the BPM’s resolution requirement of 0.5 mm.

### 4.6 Beam position measurement accuracy evaluation

The position measurement accuracy of the cavity BPM is determined by two principal factors: the position signal sensitivity and x/y directional coupling effects. Experimental characterization of the prototype BPM confirm that, under clinical operating conditions, the output signal amplitudes attains the nanovolt scale (~nV). These nanoscale signals are vulnerable to be obscured by noise sources several orders of magnitude stronger, necessitating the use of lock-in amplification methodology for reliable signal retrieval. This technology with phase-sensitive detection to extract signal components at specific reference frequencies and phases while suppressing noise at other frequencies.

An SR844 model lock-in amplifier is used for evaluating the position measurement accuracy of this prototype BPM (for technical specifications and operating procedures, refer to the instrument manual). Performance evaluation was conducted at five characteristic energy points across the proton therapy energy range (70–230 MeV), with quantitative results shown in **Table 4**. The measured position accuracy demonstrates energy dependence, ranging from 0.65 mm at 70 MeV to 0.02 mm at 230 MeV. Notably, the cavity BPM maintains sub-0.5 mm accuracy throughout most of the therapeutic energy spectrum.

**Table 4:** Energy-dependent measured proton beam position accuracy at different energy.

Beam energy (MeV)	Beam current (nA)	Position sensitivity (nV/mm)	Position accuracy (mm)
70	0.35	0.49	0.65
110	1.38	3.12	0.10
150	1.81	5.13	0.06
210	3.41	11.46	0.03
230	5.00	17.12	0.02

Additional measurements reveal that when the beam deviates within ±10 mm from the central axis, x/y directional coupling induces output signal errors less than 0.5%. These results indicate that the fundamental limitation in positional measurement accuracy stems principally from the resonant cavity’s intrinsic sensitivity characteristics.

While the current prototype utilizes cost-effective stainless steel fabrication for proof-of-principle validation, this material selection inherently constrains its detection sensitivity. Substantial performance enhancement is theoretically achievable through precision-engineered copper cavity BPM systems, thereby satisfying the requirements (≤ 0.5 mm) for real-time, non-intercepting beam monitoring applications.

## 5 Conclusion

This paper introduces an innovative cavity BPM design tailored to overcome the challenges associated with monitoring low-intensity, low-frequency clinical proton pulses, typical in cyclotron-based proton therapy systems. These systems frequently use an energy degrader to regulate beam energy, complicating real-time diagnostics due to the low beam current. Traditional cavity BPMs, which typically operate in higher modes, face limitations under such conditions. Operating in higher modes leads to weaker electromagnetic field excitation inside the cavity, reducing the sensitivity needed to detect low-current beams. Additionally, these cavities’ large transverse dimensions, necessary to match the low repetition rates, render them impractical for some clinical applications.

To overcome the limitations of traditional designs, our novel cavity BPM features an off-center rectangular resonant cavity operating in the TM110 fundamental mode. This innovative configuration significantly enhances detection sensitivity. Additionally, the cavity is filled with alumina ceramic, which increases the equivalent capacitance and lowers the resonant frequency, enabling a more compact and efficient design. As a result, the transverse dimensions of the cavity have been drastically reduced from the conventional 4236×2118 mm to a streamlined 500×250 mm, while maintaining exceptional performance.

Conventional BPMs, including ionization chambers and fluorescent screens, achieve high-precision beam position measurement. Nevertheless, these interceptive devices necessitate physical insertion of detection materials into the beam path, resulting in inevitable beam perturbation. Specifically, fluorescent screen BPMs require complete beam interception, restricting their application exclusively to beam commissioning procedures. By comparison, resonant cavity BPMs offer non-intercepting online beam position monitoring while maintaining uninterrupted beam transmission, with the additional capability of concurrent real-time proton beam energy verification.

The prototype cavity BPM has undergone comprehensive cold tests using a network analyzer. These tests indicate that the novel cavity BPM can detect proton beams with energies from 70 to 230 MeV. Measurements demonstrate energy-dependent sensitivities of 0.49 nV/mm at 70 MeV and 17.12 nV/mm at 230 MeV and with less than 0.5% crosstalk between the x and y directions over ±10 mm. Within the therapeutic energy range (70–230 MeV), experimental measurements confirm that the energy-dependent beam position accuracy remains within 0.5 mm, with the exception observed at the lowest energy point.

The performance of the novel cavity BPM is anticipated to be significantly enhanced upon realization of a fully functional prototype, thereby offering an effective technical approach for real-time, online monitoring of therapeutic proton beams.

## Data Availability

The original contributions presented in the study are included in the article/supplementary material. Further inquiries can be directed to the corresponding author.
